# Significance of Circulating and Crevicular Matrix Metalloproteinase-9 in Rheumatoid Arthritis-Chronic Periodontitis Association

**DOI:** 10.1155/2015/218060

**Published:** 2015-03-03

**Authors:** Isabela Silosi, Manole Cojocaru, Lili Foia, Mihail Virgil Boldeanu, Florin Petrescu, Petra Surlin, Viorel Biciusca

**Affiliations:** ^1^Department of Immunology, University of Medicine and Pharmacy of Craiova, 2 Petru Rares Street, 200349 Craiova, Romania; ^2^Department of Physiology, University Titu Maiorescu of Bucharest, Faculty of Medicine, 187 Calea Vacaresti Street, 004051 Bucharest, Romania; ^3^Department of Biochemistry, University of Medicine and Pharmacy of Iasi, 16 University Street, 700115 Iasi, Romania; ^4^Department of Medical Semiology, University of Medicine and Pharmacy of Craiova, 2 Petru Rares Street, 200349 Craiova, Romania; ^5^Department of Periodontology, University of Medicine and Pharmacy of Craiova, 2 Petru Rares Street, 200349 Craiova, Romania

## Abstract

In the recent years, statistically significant associations between rheumatoid arthritis (RA) and periodontal disease have been identified. Emerging as a chronic inflammatory joint disease, RA displays various features and pathogenetic events similar to chronic periodontitis (CP). The purpose of this study was to evaluate the utility of determining systemic and crevicular levels of metalloproteinase-9 (MMP-9) as potential biomarkers for association between RA and CP. A total of fifty-six patients were included in the study. The subjects were categorized into four groups as follows: healthy-control (*n* = 21), active RA (*n* = 16), CP (*n* = 14), and RA-CP association (*n* = 12). Assessment of serum and crevicular concentrations of total MMP-9 (active and pro-MMP-9) was based on ELISA technique. The results of this study showed statistically significant differences of serum MMP-9 between patients groups and control. Serum levels of MMP-9 were similar in RA and RA-CP associated patients. Gingival crevicular fluid (GCF) recorded increased MMP-9 levels in RA-CP association subjects as compared to CP. Considering that RA-CP association is characterized by a disregulation of the inflammatory response, MMP-9 may play a role in the pathogenesis of RA-CP association. MMP-9 is therefore a sensitive tool in the diagnosis and management of patients affected by this binomial association.

## 1. Introduction

The oral cavity is thought to be the mirror of the body because oral manifestations accompany many systemic diseases [1]. Periodontal disease is among the most commonly occurring infections in human, with potentially profound effects on organism health [2–4].

Some studies have reported a high incidence of rheumatoid arthritis (RA) in patients with periodontitis (P), suggesting P to be a triggering factor for RA [5, 6]. The prospective clinical trials have shown that individuals with RA are more likely to experience moderate to severe chronic periodontitis (CP) as compared to healthy subjects. The chronic inflammatory lesion that characterizes both of these disorders leads to strikingly similar consequences to the surrounding calcified and soft tissues. The clinical association between these two disorders, now demonstrated in several populations, is complex and may relate to a number of biological factors. Moreover, the intimate immune mechanisms involved in the development of the autoimmunity that leads to RA are active in the oral cavity, as well [7, 8].

Several clinical studies have indicated a potential positive association of RA with P, an infectious disease initiated by oral anaerobic bacteria. It has been documented that patients with RA are more likely to exhibit P than patients without RA [9–11]. Periodontitis is a multifactorial infection characterized by a destructive inflammatory process affecting tooth supporting tissues and resulting in periodontal pocket formation and alveolar bone resorption, which might eventually lead to tooth loss [12].

Gingival crevicular fluid (GCF) is a serum exudate that originates in the microcirculation of the gingival tissues and flows into the gingival sulcus or periodontal pocket, carrying mediators of local tissue destruction and byproducts of tissue metabolism. This oral fluid is used for detection and monitoring of certain immunomarkers concentration [13, 14].

RA is characterized by local and systemic inflammatory host responses. In addition to T and B lymphocytes, phagocytes have a crucial role in the pathogenesis of synovial inflammation, by secretion of various proinflammatory cytokines and metalloproteinases (MMPs) [15].

So far, the relationship between periodontal disease and RA is insufficiently investigated. The aim of this study was to determine whether MMP-9 measured within GCF and/or serum could act as biomarker for both these diseases and to P-associated RA, in order to unveil additional markers that connect CP with the appearance of RA.

## 2. Materials and Methods

We analyzed a 63 total number of individuals, 42 patients diagnosed with clinically active RA (*n* = 16), CP (*n* = 14), and RA-CP association (*n* = 12) and 21 healthy subjects, matched by age and gender. Following the revised classification criteria of the American Rheumatism Association [16], the subjects were investigated, diagnosed, and included into the studied groups by a rheumatologist and periodontist. The defined rheumatoid arthritis was confirmed on presence of synovitis in at least 1 large joint (score range 0–5), with a duration of at least 6 weeks (range 0-1), with positive serology for rheumatoid factor, and presence of cyclic citrullinated peptide antibodies (score range 0–3), and elevated acute-phase response (score range 0-1). All patients with CP and RA-CP had a severe generalized form of CP, while RA patients did not exhibit any comorbidities. Periodontal criteria for inclusion were as follows: at least four teeth with probing depth (PD) > 6 mm on both maxillaries, radiographic evidence of bone loss being apparent, at least 12 teeth remaining in total.

No history of medication other than anti-inflammatory drugs in the previous 6 months and no previous periodontal treatment were recorded for RA and RA-CP patients. Moreover, we excluded, from the start, the women during pregnancy or receiving hormonal or vitamin therapy.

All RA patients were assessed according to a predefined protocol including the periodontal status (visible plaque scores, bleeding scores, probing depth, and number of present teeth). For this study we obtained the approval of the Committee of Ethics and Academic and Scientific Deontology of the University of Medicine and Pharmacy from Craiova number 76/2014.

### 2.1. Sample Collection

Blood and GCF samples were collected from all subjects. Peripheral venous blood was collected into separator vacutainers and allowed to clot for 30 min at room temperature. The test tubes were centrifuged at 3000 ×g for 10 min, and serum samples were further divided into aliquots and stored at −80°C, until assessment. GCF was collected using paper strips (PerioPaper, Oraflow Inc., USA) maintained for 30 seconds in the gingival sulcus. For each subject, the fluid was collected from four teeth affected by CP, being thereafter put together. The samples volume was measured with a specific device, Periotron 8000,discarded in polypropylene tubes with 100 *μ*L PBS(phosphate-buffer saline) and stored at −20°C prior to use.

### 2.2. Immunological Investigations

The analysis of serum and GCF concentrations of total MMP-9 (active and pro-MMP-9) in patients with RA, CP, and RA-CP association was based on a quantitative sandwich ELISA, using the MMP-9 assay kit (R&D Systems, USA).

### 2.3. Statistical Analysis

The clinical parameters and continuous variables were expressed as mean value ± standard deviation (SD), based on the subject as the statistical unit. After multiple comparisons test between groups with ANOVA (statistically significant differences when *P* < 0.01), comparisons of the serum and crevicular levels of parameters between each test group and control were performed using Student's *t*-test. Differences between groups were considered to be statistically significant when *P* recorded values were <0.05.

## 3. Results

The demographic and clinical data of the studied individuals are presented in Table 1. The control subjects were similar in age and gender to investigated patients but were clearly distinct in terms of the measured parameters.

The results of ANOVA test showed significant statistical differences between groups, *P* < 0.001.

Comparing each test group with the control, our results point out that GCF and serum levels of MMP-9 in patients with RA were significantly higher than in healthy persons. In CP patients, GCF MMP-9 levels and the magnitude of MMP-9 expression in serum were significantly greater than in control group. Moreover, RA-CP group had significantly higher GCF and serum MMP-9 when compared to control group (Table 2).

Gingival fluid MMP-9 levels in RA-CP patients were significantly elevated compared to CP and RA subjects. Serum MMP-9 concentrations were significantly higher in patients with RA-CP association than in CP but did not differ significantly from RA (Table 2).

In our study, the incidence of women with RA diagnosis was 75% (sex ratio 4 M : 12 F) and 77% in RA-CP group (sex ratio 9 F : 3 M), data which are congruent with the results of other studies showing female predominance in RA [17]. Considering the magnitude of our batch of subjects in the present study, we did not focus on a statistical gender analysis, the possible groups males: females being rather limited.

## 4. Discussions

Periodontal disease is one of the most prevalent inflammatory disorders, modulated by several factors, among which, that include some members of metalloproteinases family, derived from the host upon tissue distruction. Every MMP has distinct potential on extracellular matrix degradation, jeopardizing dental units life and metabolism and resulting in degenerative events at the oral level. Whereas the magnitude of these inducible enzymes is rather low in normally healthy periodontium, augmented concentrations of the MMPs can be recorded in injured and inflammatory conditions of periodontal tissues. In particular, MMP-9 has been reported as specifically breaking down type IV collagen, which is an extensive structural component of basement membrane [18].

Recent studies have related periodontal disease to several immune, hormonal, or connective tissue metabolism impairments, of which rheumatoid arthritis is of circumstantial significance, since, despite the different etiology, its pathogeny shares a similar pattern, concerning the breakdown of tissue architecture, with that of chronic periodontitis.

Thus, both are destructive and inflammatory diseases, characterized by accumulation and persistence of inflammatory infiltrate in local lesions. Patients with RA and seropositive for RF and the anti-CCP antibodies are more likely to develop CP, compared to patients without these markers at systemic level and presenting a high degree of periodontal tissue destruction [5, 7].

Therefore, the main objective of this study was to quantify the activity of MMP-9 in associated inflammatory conditions and to determine whether systemic or crevicular levels of this metalloproteinase could be useful as biomarkers for RA and CP association.

Elevated serum and GCF MMP-9 levels were observed for CP, RA, and RA-CP patients as compared to healthy subjects. In CP group MMP-9 concentrations were significantly increased in serum and in GCF as compared to controls. The degradation of collagen fibers and other extracellular matrix components in inflammatory diseases, such as periodontitis, results from the activity of matrix metalloproteinases which originate in various cellular types, from polymorphonuclear leucocytes and macrophages, to bone, epithelial, endothelial cells, and fibroblasts [18]. Periodontal microorganisms induce host response with increased release of MMP8 and MMP-9 in the periodontal pockets. The microbial enzymes and host matrix metalloproteinases (MMP8 and MMP-9) appear to play important roles in both conditions, RA and CP [7, 13, 19].

MMP-9 or gelatinase B is a multidomain enzyme functioning in acute and chronic inflammatory and neoplastic diseases. MMP-9 is essential for initiating the osteoclastic resorption process by removing the collagenous layer from the bone surface before demineralization can start [20].

The role of MMPs as inflammatory mediators in the pathogenesis of periodontal breakdown was reported by numerous authors [21–26]. The central question is whether these MMPs can influence the outcome of inflammation and if so, what is the underlying mechanism. MMPs can influence the progression of various inflammatory conditions and are important for periodontal tissue destruction [27, 28].

Based on our data, and confirmed by other researchers, elevated systemic and local levels of the investigated parameter MMP-9 suggest its value as a reliable inflammatory biomarker for the periodontal injury and RA, with a distinct role in the modulation of oral or synovial inflammatory response [29].

Inflammatory conditions are almost always characterized by dysregulated, often increased, MMPs activities. The patients with periodontitis respond to bacterial invaders by mobilizing their defensive cells and releasing proinflammatory cytokines like interleukin-1*β*, tumor necrosis factor-*α*, and interleukin-6, which ultimately causes tissue destruction by stimulating the production of collagenolytic enzymes like matrix metalloproteinases [30].

In our study, crevicular MMP-9 recorded higher concentrations in patients with CP than in controls (*P* < 0.01). These results are in agreement with previous findings, significant differences being also observed in the levels of MMP-9 in GCF of CP patients when compared to healthy subjects [31, 32].

During chronic periodontitis, in response to endotoxin derived from periodontal pathogens, several osteoclast-related mediators target the destruction of alveolar bone and supporting connective tissue. Major drivers of this aggressive tissue destruction are MMPs, cathepsins, and other osteoclast-derived enzymes [33, 34]. Gelatinase B was implicated in bone loss [35]. MMP-9 could be actively involved in the extracellular matrix degradation in apical periodontitis lesions [36]. Crevicular MMP-9 levels decrease after periodontal therapy [37].

The expression and activity of MMPs in noninflamed periodontium is low, but it is drastically enhanced to pathologically elevated levels due to the dental plaque and infection-triggered periodontal inflammation. However, other studies suggest that MMPs can also exert anti-inflammatory effects in defence of the host, by modulating anti-inflammatory cytokines and chemokines, as well as by regulating apoptotic and immune responses [28]. Prior studies also suggested that specific species of bacteria implicated as pathogens in periodontitis may be involved in the pathogenesis of RA [3, 38]. Patients with RA displayed high serum concentrations of tissue-degrading metalloproteinases [39]. We found that in RA patients the crevicular and serum MMP-9 levels were higher than those detected in healthy subjects. These data suggest that once these MMPs are fully activated, they contributed to the cartilage destruction in RA. Elevated levels of MMPs were found in synovial fluid of RA patients. Augmented systemic MMP-9 levels represented the situation in the inflamed joint. MMP-9 is likely to be involved in degradation of joint collagen [22, 40].

Involvement of MMP-9 in periodontal remodeling process is known but is insufficiently clarified known but is insufficiently clarified on RA-CP association. Like RA, adult periodontitis is a chronic inflammatory disorder in which the accumulation of immune cells leads to local production of proinflammatory cytokines such as tumor necrosis factor, interleukin-1, MMPs, and prostaglandins, which results in tissue swelling and degradation [41].

We found that in patients suffering of periodontal-rheumatoid arthritis association, serum MMP-9 concentrations are significantly higher than in CP, whereas crevicular MMP-9 levels were distinctly elevated than in CP patients, as well. Those results show that in the case of patients with RA-CP association the inflammatory reaction is more profound, probably because their immune system is continuously activated by periodontal pathogens and other unknown factors for RA. The chronic inflammation and disorder caused by periodontitis may predispose the individuals to the development of rheumatoid arthritis, disease triggering itself a substantial inflammatory feedback [42].

While the etiology of these two diseases may differ, the underlying pathogenic mechanisms are remarkably similar and it is possible that individuals manifesting both periodontitis and RA may suffer from a unifying underlying systemic dysregulation of the inflammatory response [43, 44]. There is almost universal acceptance that a variety of cytokines and matrix metalloproteinases are upregulated and intimately involved in the pathogenesis of both periodontitis and RA; many of these effector molecules appear to be common to both diseases [43, 45]. MMP-9 upregulation can be highly induced by a wide range of agents. These agents include growth factors and other cytokines [46].

The levels of MMP-9 were significantly higher in the group with severe compared with mild disease. These observations suggest that measurement of serum MMP-9 of RA-CP patients could be useful in the disease progress monitoring. It was suggested that circulating MMP-9 levels are associated with disease severity in RA disease [47].

Our findings provide evidence that MMP-9 has important roles in different phases of disease and that measurement of serum MMP-9 could be of clinical value for identifying patients at high risk for progression. We also suggest that MMP-9 has a role in pathogenesis of RA-CP association, inflammation-induced connective tissue degradation involved being very probably partly mediated by MMP-9 signaling, and, therefore, this metalloproteinase could be used as a noninvasive serum marker for inflammation in this pathology. Moreover, considering that matrix metalloproteinases have been recommended as drug targets in infections caused by Gram-negative bacteria [48], we assume that, very probably, MMP-9 could be a useful marker for the follow-up of the antimetalloproteinase therapy in RA-CP association. Our data sustain the concept that periodontitis could be a causal factor in the initiation and maintenance of the autoimmune inflammatory response that occurs in RA. The main answer could be found in changes of the parameters not only on the systemic level, but also on the feedback that appears within gingival crevicular fluid, this exudate that precisely reflects the biologic events in the periodontium [49, 50]. The present results indicate that serum and GCF MMP-9 levels are significantly elevated, sustaining the involvement of MMPs proteins in these autoimmune conditions. In the context of our findings, it could be suggested that MMP-9 participates in the very complex chain of mediators that modulate the inflammation in the studied diseases. Only by understanding the mechanism by which MMPs exert their function, they could contribute to the development of new and effective drug targets.

## 5. Conclusions

Within the limits of data provided by this study, we can conclude that the periodontitis-associated rheumatoid arthritis may be a reflection of a common underlying disregulation of the inflammatory response in these individuals.

Crevicular MMP-9 levels, significantly higher in patients with RA-CP association than in CP, reinforce that the inflammatory reaction is more intense, perhaps because their immune system is continuously activated by periodontal pathogens.

Matrix metalloproteinase 9 may represent a key modulator of the biological pathogenic mechanisms triggered in the periodontium by the binomial CP-RA, the levels of this marker being therefore important, for the identification of patients affected by this association.

The limits of this study related in particular to the small number of patients included in the study, which is why we continued with the expansion of the group and reconsideration results.

## Figures and Tables

**Table 1:** The demographic and clinical data of the studied individuals.

Subjects	Number	Age range (years)	Sex ratio (M : F)

Control	21	35–58	7 : 14
Chronic periodontitis (CP)	14	39–68	6 : 8
Associations Rheumatoid arthritis-chronic periodontitis (RA-CP)	12	38–62	3 : 9
Rheumatoid arthritis (RA)	16	38–69	4 : 12

**Table 2:** MMP-9 levels of crevicular and serum concentrations in RA, RA-CP, CP, and healthy groups and statistical significance comparison between groups.

Parameter (mean ± SD)	Control group (*N* = 21)	RA group (*N* = 16)	CP group (*N* = 14)	RA-CP group (*N* = 12)
Serum MMP-9 (ng/mL)	34.12 ± 15.4	498.12 ± 143.72 *P* < 0.001^*^	54.81 ± 25.93 *P* < 0.01^**^	640.18 ± 247.76 *P* < 0.001^***^ *P* < 0.001^#^ *P* > 0.05^##^

GCF MMP-9 (ng/mL)	25.06 ± 11.9	44.16 ± 18.96 *P* < 0.001^*^	42.31 ± 19.92 *P* < 0.01^**^	58.67 ± 14.23 *P* < 0.001^***^ *P* < 0.05′′ *P* < 0.05^′′ ′′^

^*^Significant statistical differences between RA and control group for serum and GCF MMP-9.

^**^Significant statistical differences between CP and control group for serum and GCF MMP-9.

^***^Significant statistical differences between RA-CP and control group for serum and GCF MMP-9.

^#^Significant statistical differences between RA-CP and CP group for serum MMP-9.

′′Significant statistical differences between RA-CP and CP group for GCF MMP-9.

^′′ ′′^Significant statistical differences between RA-CP and RA group for GCF MMP-9.

^##^No significant statistical differences between RA-CP and RA group for serum MMP-9.
